# Human Intestinal Lumen and Mucosa-Associated Microbiota in Patients with Colorectal Cancer

**DOI:** 10.1371/journal.pone.0039743

**Published:** 2012-06-28

**Authors:** Weiguang Chen, Fanlong Liu, Zongxin Ling, Xiaojuan Tong, Charlie Xiang

**Affiliations:** 1 State Key Laboratory for Infectious Diseases Diagnostics and Treatment, the First Affiliated Hospital, College of Medicine, Zhejiang University, Hangzhou, China; 2 Department of Anus and Intestine, the First Affiliated Hospital, College of Medicine, Zhejiang University, Hangzhou, China; University of Bari & Consorzio Mario Negri Sud, Italy

## Abstract

Recent reports have suggested the involvement of gut microbiota in the progression of colorectal cancer (CRC). We utilized pyrosequencing based analysis of 16S rRNA genes to determine the overall structure of microbiota in patients with colorectal cancer and healthy controls; we investigated microbiota of the intestinal lumen, the cancerous tissue and matched noncancerous normal tissue. Moreover, we investigated the mucosa-adherent microbial composition using rectal swab samples because the structure of the tissue-adherent bacterial community is potentially altered following bowel cleansing. Our findings indicated that the microbial structure of the intestinal lumen and cancerous tissue differed significantly. Phylotypes that enhance energy harvest from diets or perform metabolic exchange with the host were more abundant in the lumen. There were more abundant Firmicutes and less abundant Bacteroidetes and Proteobacteria in lumen. The overall microbial structures of cancerous tissue and noncancerous tissue were similar; howerer the tumor microbiota exhibited lower diversity. The structures of the intestinal lumen microbiota and mucosa-adherent microbiota were different in CRC patients compared to matched microbiota in healthy individuals. Lactobacillales was enriched in cancerous tissue, whereas *Faecalibacterium* was reduced. In the mucosa-adherent microbiota, *Bifidobacterium, Faecalibacterium*, and *Blautia* were reduced in CRC patients, whereas *Fusobacterium*, *Porphyromonas*, *Peptostreptococcus*, and *Mogibacterium* were enriched. In the lumen, predominant phylotypes related to metabolic disorders or metabolic exchange with the host, Erysipelotrichaceae, Prevotellaceae, and Coriobacteriaceae were increased in cancer patients. Coupled with previous reports, these results suggest that the intestinal microbiota is associated with CRC risk and that intestinal lumen microflora potentially influence CRC risk via cometabolism or metabolic exchange with the host. However, mucosa-associated microbiota potentially affects CRC risk primarily through direct interaction with the host.

## Introduction

Colorectal cancer (CRC) is one of the most common malignant tumor type in the world. One of the important factors associated with CRC is the intestinal microbiota [1]. The human gastrointestinal tract harbors approximately 1000 species of bacteria totaling 10^14^ cells, which is more than 10-fold the number of eukaryotic human cells [2]. In addition to influencing host nutrition via metabolism, the intestinal microbiota affects the human body by controlling epithelial proliferation and differentiation, influencing the development of the immune system and protecting against pathogens [3].

Accumulating evidence suggests that the gut microbiota is closely correlated with the progression of colorectal cancer [1], [4]. Wei *et al*. found an increase of *Ruminococcus obeum* and *Allobaculum*-like bacteria in the feces of rats developing precancerous mucosal lesions [5]. An increase of *Prevotella* was reported in CRC patients [6]. Wang *et al*. found a reduction in butyrate-producing bacteria in the feces of CRC patients [7], indicating the benefit of bacterial metabolites.

However, the mucosa-associated microbiome in intestinal tissue differs from the lumen [8], and these microbes also potentially play important roles. Marchesi and coworkers analyzed the bacterial 16S rDNA sequences of six CRC patients and determined that probiotic bacteria such as *Coriobacteria* were enriched in tumor tissue by analyzing the bacterial 16S rDNA sequences of six CRC patients [9], suggesting that probiotics potentially play a special role in CRC progression. *Fusobacterium nucleatum* found in colon cancer tissue was reported to be closely associated with CRC [10]. However, the exact composition of intestinal microbiota and its function in CRC progression are remain unknown because the overall structure of microbiota in CRC patients has not been completely elucidated.

Bacteria or components of bacteria function by direct interaction with the host or indirect co-metabolism or metabolic exchange with the host. Van der Waaij *et al*. found that commensal bacteria live in the intestinal lumen suspension and have no direct contact with epithelial cells [11]. We hypothesized that the mucosa-associated microbiota primarily function by directly interacting with the host and that intestinal lumen (i.e., stool) microbiota principally act through cometabolism or metabolic exchange. In this study we performed pyrosequencing of 16S rRNA genes in order to analyze the overall structure of microbiota in patients with CRC and in healthy controls. We studied the microbiota of the intestinal lumen, cancerous tissue, and matched noncancerous normal tissue. In addition, we examined the mucosa-adherent microbial composition by using rectal swab samples because the structure of the tissue-adherent bacterial community is potentially altered by following bowel cleansing [12]. Moreover, we attempted to identify key bacterial phylotypes or potential biomarkers that potentially play important roles in CRC development.

## Materials and Methods

### Patients and Control Groups

A total of 46 patients with CRC 37–88 years of age were selected from the First Affiliated Hospital, College of Medicine, Zhejiang University, China. We gathered the following samples from these patients: [21 stool (stp) samples, 32 gut swab (swp) samples and 27 of each of cancerous tissue (cat), matched paracancerous tissue 2–5 cm from the cancerous tissue (pa2t), and matched paracancerous tissue 10–20 cm from the cancerous tissue (pa10t)]. The stool, rectal swab, and tissue samples were not collected from all CRC patients for the following reasons: watery stool, stool too thin to collect; samples stored too long at room temperature; or the patient felt uncomfortable during the collection of rectal swab samples. Additionally, 56 healthy volunteers who met the requirements of having matched gender and similar age with the samples of CRC patients, and who exhibited no colonic adenomas were selected as controls; 22 stool samples (stc) and 34 swabs (swc) were collected from these volunteers (Table 1). We defined microbiota of tissue, stool, and swab as tissue microbiota, lumen microbiota, and mucosa-adherent microbiota, respectively. We defined both tissue microbiota and mucosa-adherent microbiota as mucosa-associated microbiota. No study subjects had diabetes, infectious diseases, or particular diets. And the BMI of all subjects was between 20 and 24. This study was approved by the Ethics Committee of the First Affiliated Hospital, College of Medicine, Zhejiang University, China; documented informed consent was obtained from all study participants.

**Table 1:** Summary information of samples.

	Healthy volunteers(56)		CRC patients(46)		
Sample	Swab	Stool	Swab	Stool	Tissue
No.	34	22	32	21	27×3
Male/female	20/14	11/11	21/11	11/10	14/13
Age (mean,range)	56(42–77)	64(37–84)	65(37–86)	64(37–78)	61(37–81)

32 swabs samples, 21 stool samples and 27 sets of tissue samples were collected from 46 CRC patients.

### Sample Collection and DNA Extraction

None of the subjects were taking medications at the time of sample collection, nor had they used antibiotics within at least one month of sample collection. Swab and fecal samples were collected from each subject prior to bowel cleansing. During surgery, intestinal samples were collected from cancerous tissue and paracancerous tissue (i.e., 2–5 cm and 10–20 cm from the cancerous tissue, respectively). All samples were frozen and stored at −80°C until further use.

Genomic DNA was extracted from tissue and swab samples by using the QIAamp DNA Mini Kit (Qiagen, Hilden, Germany) according to the manufacturer’s instructions with minor modifications. Bacterial cells in swabs were dislodged by vigorous agitation in 1 ml PBS. The cells were pelleted by centrifugation at 17,000 g for 10 min. The pellets were resuspended in 80 µl enzyme solution (22.5 mg lysozyme powder [catalog no.L6876, Sigma] and 40 units mutanolysine [catalog no. M9901, Sigma] dissolved in 80 µl TE per sample) [13], and 100 mg of zirconium beads (0.1 mm) were added. The mixtures were agitated in a mini-bead beater (FastPrep, Thermo Electron Corporation, USA) three times for 40 s each time, and then incubated at 37°C for 40 min [14]. Subsequent steps were performed according to manufacturer’s recommendations. Intestinal tissues were extensively rinsed with sterile water, homogenized in 80 µl enzyme solution using an electric homogenizer (PRO Scientific, Oxford, Connecticut, USA), incubated at 37°C for 40 min, and then completely lysed for 1–3 hours at 56°C in ATL buffer and proteinase K. The 70°C incubation step was extended from 10 minutes to 30 minutes [8]. Stool bacterial genomic DNA was extracted using QIAamp DNA Stool Mini Kit with the same modifications as listed above.

### Pyrosequencing

PCR amplification of the V1-V3 region of bacterial 16 S rDNA was performed using universal primers (27F 5′-AGAGTTTGATCCTGGCTCAG-3′, 533R 5′-TTACCGCGGCTGCTGGCAC-3′) incorporating the FLX Titanium adapters and a sample barcode sequence. The cycling parameters were as follows: 5 min initial denaturation at 95°C; 25 cycles of denaturation at 95°C (30 s), annealing at 55°C (30 s), elongation at 72°C (30 s); and final extension at 72°C for 5 min. Three separate PCR reactions of each sample were pooled for pyrosequencing. The PCR products were separated by 1% agarose gel electrophoresis and purified by using the QIAquick Gel extraction kit (Qiagen). Equal concentrations of amplicons were pooled from each sample. Emulsion PCR and sequencing were performed according to the manufacturer’s recommendations [15].

All pyrosequencing reads were filtered according to barcode and primer sequences. The resulting sequences were further screened and filtered for quality and length. Sequences that were less than 150 nt, contained ambiguous characters, contained over two mismatches to the primers, or contained mononucleotide repeats of over six nt were removed [16]. A total of 808,008 high-quality sequences were produced, accounting for 80.8% of valid sequences according to barcode- and primer-sequence filtering.

### Bioinformatic Analysis

The high-quality sequences were assigned to samples according to barcodes. Sequences were aligned in accordance with SILVA alignment [17], [18] and clustered into operational taxonomic units (OTUs). OTUs that reached 97% similarity level were used for diversity (Shannon), richness (Chao), Good’s coverage, and Rarefaction curve analysis by using Mothur (version 1.5.0) http://schloss.micro.umass.edu/ [19]. Taxonomical assignments of OTUs exhibiting 97% similarity were performed by using Mothur in accordance with SILVA 106 at 80% confidence level.

The heatmap was constructed by using the heatmap 2 function of the R gplots package and genus information of seven groups [20]. Unweighted UniFrac distance metrics analysis was performed using OTUs for each sample [21], [22], and principal component analysis (PCA) was conducted according to the matrix of distance. To select OTUs that exhibited significance in the structural segregation between groups, a parametric Partial least squares Discriminant Analysis (PLS-DA) model was generated by using Simca-P+12.0 (http://www.umetrics.com/). PLS-DA is utilized in metabolomics, metagenomics and microarray analysis, and OTUs with variable importance in projection (VIP)>1 were considered to be important contributors to the model [5], [23], [24], [25].

### Statistical Analysis

The Mann-Whitney test, *t*-test, and one-way ANOVA test were performed using SPSS version 19.0 for Windows.

### Data access

The 16 S sequence data generated in this study were submitted to the GenBank Sequence Read Archive accession number SRP009633.

## Results

### Characteristics of Pyrosequencing Results

A total of 808,008 high-quality sequences were produced in this study, with an average of 4253 sequences per sample. Summary information is shown in Table 2, and detailed characteristics of each sample are found in Table S1.

**Table 2:** Pyrosequencing data summary.

	cat(n = 27)	pa2t(n = 27)	pa10t(n = 27)	stc(n = 22)	stp(n = 21)	swc(n = 34)	swp(n = 32)
Sequences	3878±796	4734±904	4564±1391	4197±468	3919±516	4349±451	4055±566
OTUs	360±117	426±108	438±84	397±68	407±60	422±66	411±68
Chao	838±312	1017±218	944±267	962±167	943±130	963±184	931±202
Shannon	3.77±0.67*	4.04±0.45	4.13±0.40*	3.70±0.54	3.89±0.49	3.96±0.44	3.98±0.59

* Shannon index between group cat and pa10t was statistically significant different (*P* = 0.012) (t-test). The number of OTUs, richness estimator Chao, and diversity estimator Shannon were calculated at 3% distance.

The estimators of community richness (Chao) and diversity (Shannon) are shown in Table 2. There were statistically significant differences of Shannon indexes between groups cat and pa10t (3.77±0.67 vs. 4.13±0.40, *P* = 0.012), demonstrating the significantly higher diversity found in noncancerous normal tissues (i.e., those 10–20 cm from cancerous tissues) compared to cancerous tissues. Detailed characteristics of each sample are listed in Table S1. The rarefaction analysis of seven groups shown in Figure S1 indicates that more phylotypes would most likely be detected after exploring larger number of sequences. The Good’s coverage of each group was over 97%, indicating that the 16 S rDNA sequences identified in these groups represent the majority of bacteria present in the samples of this study.

### Microbial Structures of Intestinal lumen and Cancerous Tissue Differed Significantly

**Figure 1:**
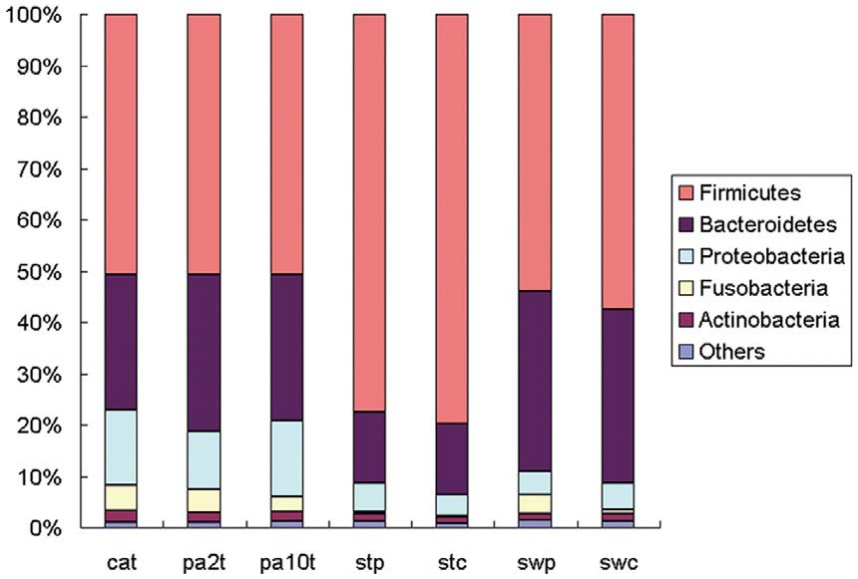
Relative abundance of bacterial phyla in microbiota of seven groups of samples. “Others” represents the unclassified bacteria, Chloroflexi, Deferribacteres, Chlorobi, Deinococcus-Thermus, Acidobacteria, Planctomycetes, Lentisphaerae, Spirochaetes, Synergistetes, Tenericutes, Verrucomicrobia and Cyanobacteria. The first eight phyla were not apparent in stool samples, and the first four phyla were not apparent in swab samples.

We studied the stool, rectal swab, and tissue microbiota of patients with CRC and the stool and rectal swab bacterial communities of healthy individuals. The overall microbiota structure for each group at the phylum level is shown in Figure 1. The dominant phyla of all groups were Firmicutes, Bacteroidetes, and Proteobacteria. There were 17 phyla and 13 phyla in tissue and swab samples, respectively, and only 9 phyla in stool samples. The phylum-specific relative abundance of microbiota sequences revealed that swab microbiota exhibited a closer similarity to tissue. The heatmap according to bacterial genus level also demonstrated the same phenomenon (Figure S2).

**Figure 2:**
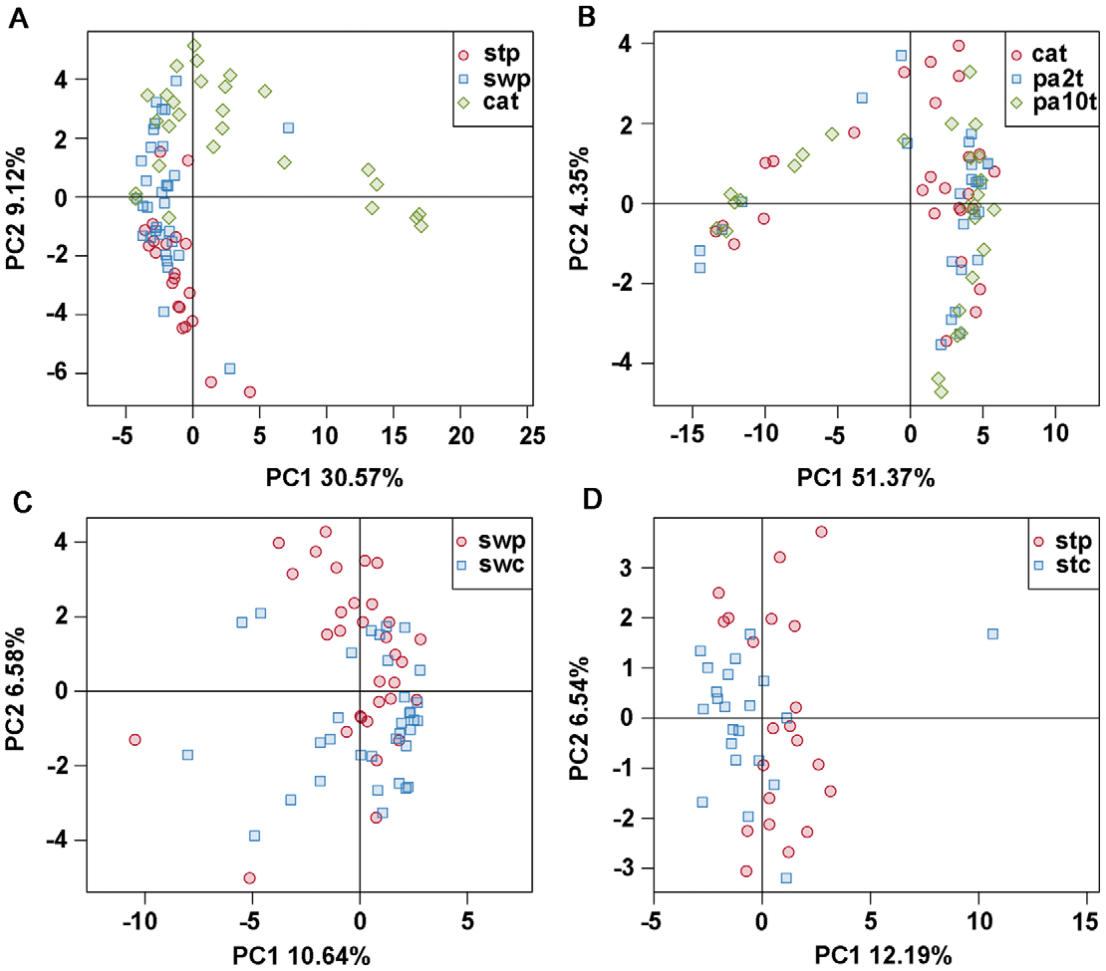
PCA plots based on unweighted Unifrac metrics. Each symbol represents a sample. (A) group cat, stp and swp; (B) group cat, pa2t and pa10t; (C) group swp and swc; (D) group stp and stc.

To compare the overall microbiota structure in patients with CRC, the unweighted Unifrac distance matrix was calculated based on the OTUs of each sample [26]. The results of PCA based on distance exhibited a significant difference in bacterial structure in intestinal lumen (i.e., stool). Furthermore, cancerous tissue, and mucosa-adherent microbiota (i.e., swab) overlapped with some lumen and tissue microbiota, as demonstrated by the first two principal component scores, that accounted for 30.57% and 9.12% of total variations (Figure 2A).

**Figure 3:**
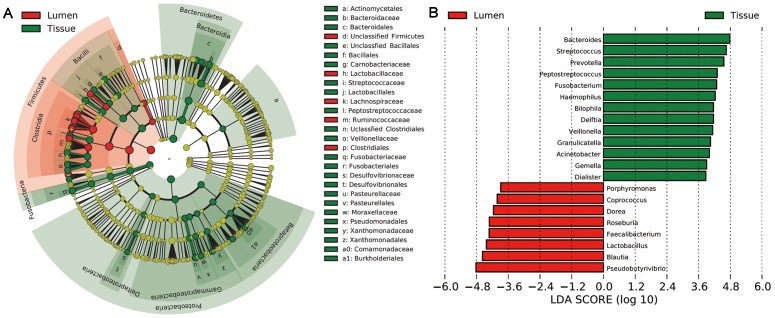
Different structures of intestinal lumen and cancerous tissue microbiota. (A) Taxonomic representation of statistically and biologically consistent differences between cancerous tissue and intestinal lumen. Differences are represented by the color of the most abundant class (Red indicating cancerous tissue, yellow non-significant and green intestinal lumen). The diameter of each circle’s diameter is proportional to the taxon’s abundance. (B) Histogram of the LDA scores for differentially abundant genera. Cladogram was calculated by LEfSe, a metagenome analysis approach which performs the linear discriminant analysis following the Wilcoxon Mann-Whitney test to assess effect size of each differentially abundant taxon or OTU; the cladogram is displayed according to effect size.

There were significant variations in the composition of intestinal lumen and cancerous tissue at different bacterial levels. A cladogram representation of the structure of tissue and lumen microbiota and their predominant bacteria was performed by LEfSe is shown in Figure 3 [27]; the greatest differences in taxa between the two communities are displayed. Pyrosequencing data demonstrated that a greater number of phyla were present in tissue compared to lumen. The three dominant phyla–Firmicutes (50.82% vs. 77.59%, *P*<0.001), Bacteroidetes (26.37% vs. 13.68%, *P* = 0.002), and Proteobacteria (14.51% vs. 5.57%, *P* = 0.004)–all exhibited statistically significant differences between cancerous tissue and intestinal lumen. Fusobacteria (4.97% vs. 0.47%, *P*<0.001) and Synergistetes (0.14% vs. 0%, *P* = 0.002) also differed between groups. There were 26 statistically significant differences between cancerous tissue and intestinal lumen at the family level. The relative abundance of Bacteroidaceae (16.9% vs. 8.3%, *P*<0.001), Streptococcaceae (10.2% vs. 2.8%, *P* = 0.0029), Fusobacteriaceae (4.57% vs. 0.47%, *P*<0.001), Peptostreptococcaceae (4.07% vs. 0.89%, *P*<0.001), Veillonellaceae (2.87% vs. 0.68%, *P* = 0.004), and Pasteurellaceae (2.25% vs. 0.007%, *P*<0.001) were significantly higher in cancerous tissue compared to the intestinal lumen. There was a significantly lower level of Lachnospiraceae (17.1% vs. 46.7%, *P*<0.001), Ruminococcaceae (4.24% vs. 13.3%, *P*<0.001), and Lactobacillaceae (0.02% vs. 2.88%, *P*<0.001) in cancerous tissue compared to the intestinal lumen (Table S2).

The microbial composition was also significantly different at the genus level, with 43 significantly different genera between cancerous tissue and intestinal lumen. *Bacteroides*, *Streptococcus*, *Prevotella*, *Fusobacterium*, *Peptostreptococcus*, *Haemophilus*, *Gemella*, *Veillonella*, *Granulicatella*, *Morganella*, and *Porphyromonas*, which constitute over 1% of the total bacteria in cancerous tissue, exhibited a relatively higher abundance in cancerous tissue. *Pseudobutyrivibrio*, *Blautia*, *Lactobacillus*, *Roseburia*, *Dorea* and *Coprococcus*, constituting which constitute over 1% of the total bacteria in stool, were relatively more abundant in intestinal lumen compared to in cancerous tissue. Additional information regarding the differences between lumen microbiota and cancerous tissue microbiota can be found in Table S2.

### Bacterial Community in Cancerous Tissue and Matched Noncancerous Normal Tissue

Although lower diversity (Shannon) was observed in microbiota of cancerous tissues (Table 2), the microbial communities of tumor and matched noncancerous normal tissues were similar (Figure 1, Figure S2). According to unweighted Unifrac PCA analysis, the microbial communities of cancerous tissue and noncancerous tissue are similar according to PC1 and PC2 (51.37% and 4.35% explained variance, respectively) (Figure 2B), indicating that there are not marked differences in the microbial composition of tumor and noncancerous tissue.

A taxonomy-based comparison was performed to determine the differences between the microbiota of tumor and noncancerous tissue. There were 12, 17, and 14 phyla and 169, 198, and 198 genera in the microbiotas of cat, pa2t, and pa10t, respectively. This was confirmed by Shannon (diversity) analysis. No statistically significant differences were observed between the microbial communities of cancerous and noncancerous tissue at the phylum level. Alphaproteobacteria, which constitute less than 1% of total bacteria in both pa2t and pa10t, were most prevalent in cat. Fewer *Ochrobactrum* genus members were present in pa2tcompared to cat. The Bacilli class was highly enriched in cat compared to pa10t. However, genus *Bacillus*, to which Bacilli belong, was less prevalent in cat. The Ruminococcaceae family was significantly lower in cat compared to pa10t. Genus *Faecalibacterium*, affiliated with Ruminococcaceae, was also highly enriched in pa10t compared to cat. Genera *Paraprevotella*, *Parabacteroides, Phascolarctobacterium*, *Acidocella*, and *Methylobacterium* exhibited low abundance; however, they were all statistically enriched in pa10t compared to cat. Moreover, the relative abundance of bacteria in the samples increased or decreased gradually in correlation with the distance from the cancerous tissue (Table 3).

**Table 3:** Phylotypes significantly different between cat and pa2t or cat and pa10t.

Taxonomic Rank		cat(%)	pa2t(%)	p value#	pa10t(%)	p value*
genus	*Ochrobactrum*	0.026	0.054	0.035	0.116	
genus	*Paraprevotella*	0.002	0.011		0.012	0.013
genus	*Phascolarctobacterium*	0.154	0.303		0.336	0.045
genus	*Parabacteroides*	0.536	0.687		0.862	0.048
family	*Ruminococcaceae*	4.24	7.12		8.28	0.031
genus	*Faecalibacterium*	1.68	3.20		4.20	0.032
class	*Bacilli*	14.56	7.31		4.82	0.015
genus	*Bacillus*	0	0.040		0.016	0.001
class	*Alphaproteobacteria*	0.136	0.250	0.017	0.725	0.024
genus	*Methylobacterium*	0.021	0.025		0.048	0.032
genus	*Acidocella*	0.002	0.020		0.052	0.029

Statistical analysis was performed by Mann-Whitney test. Data of cat, pa2t and pa10t were relative abundance (percentage) of all sequences in each group.

# P value between cat and pa2t.

* P value between cat and pa10t. P value had no statistically significant difference (≥0.05) were not shown.

The metagenome analysis approach LefSe was applied to identify the key phylotypes responsible for the difference between cat and pa10t. Bacilli (main component Lactobacillales), which was enriched in cat, and *Phascolarctobacterium*, Ruminococcaceae (main component *Faecalibacterium*), which were enriched in pa10t were the dominant phylotypes that contribute to the difference between the microbiota of cancerous tissue and noncancerous tissue.

### Mucosa-adherent Microbiota in CRC Patients and Healthy Individuals

Because the microbial composition may be changed by bowel cleansing prior to surgery, mucosa-adherent bacteria were studied in samples collected on rectal swabs. As expected, the microbial structure was somewhat different compared to tissue (Figure 1, Figure 2A) and was similar to intestinal lumen (some samples overlapped on PCA plots) because of the unavoidable feces on the swab samples.

**Figure 4:**
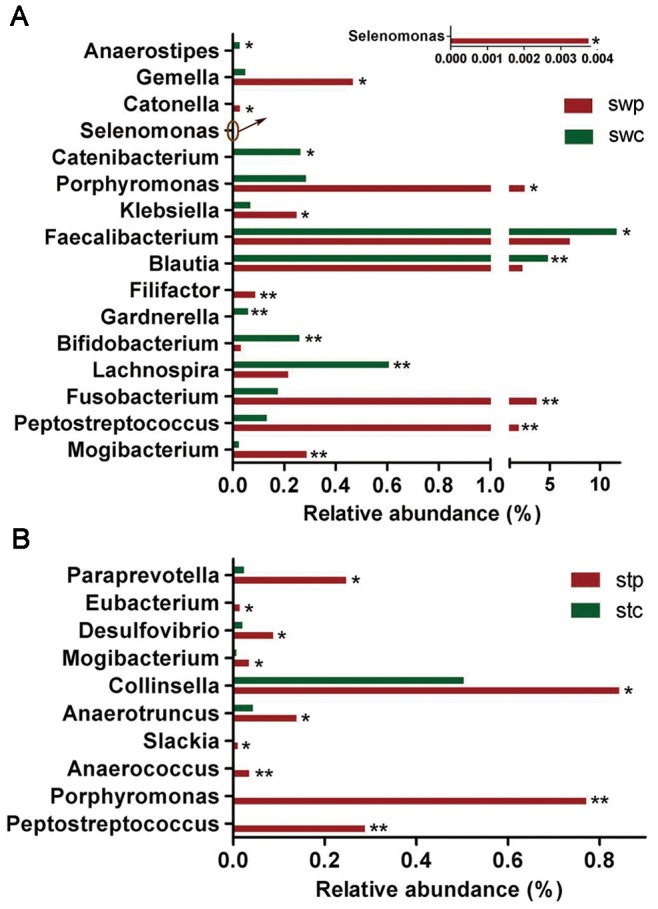
Relative abundance of significantly different genera between CRC patients and healthy controls. (A) Genera different between swp and swc. (B) Genera differing between stp and stc. The Mann-Whitney test was used to evaluate the importance of comparisons between indicated groups. *P<0.05, **P<0.01.

Unweighted Unifrac PCA based on the relative abundance of OTUs for each sample demonstrated a separation between CRC patients and healthy individuals using PC1 and PC2 (10.64% and 6.58% of explained variance, respectively) (Figure 2C). The families Porphyromonadaceae (3.86% vs. 1.41%, *P* = 0.045), Fusobacteriaceae (3.72% vs. 0.18%, *P* = 0.045), and Peptostreptococcaceae (2.13% vs. 0.66%, *P* = 0.03) were enriched in CRC patients, yet Bifidobacteriaceae (0.03% vs. 0.32%, *P*<0.001) and Alcaligenaceae (0.39% vs. 0.63%, *P* = 0.03) were reduced in CRC patients. Genera *Fusobacterium* (Fusobacteriaceae), *Porphyromonas* (Porphyromonadaceae), *Peptostreptococcus* (Peptostreptococcaceae), *Gemella*, *Mogibacterium,* and *Klebsiella* were enriched in CRC patients. *Filifactor*, *Catonella* and *Selenomonas* were absent from healthy individuals. *Faecalibacterium*, *Blautia*, *Lachnospira*, *Bifidobacterium* (Bifidobacteriaceae) and *Anaerostipes* were reduced in CRC patients, and *Catenibacterium* and *Gardnerella* (Bifidobacteriaceae) were absent from CRC patient samples (Figure 4).

*Porphyromonas* (affiliated with Porphyromonadaceae), *Fusobacterium*, *Peptostreptococcus,* and *Mogibacterium* were enriched in CRC patients, whereas *Faecalibacterium*, *Blautia*, and *Bifidobacterium* were depleted in these patients. According to LefSe analysis, these are the key phylotypes that contribute to the structural segregation of mucosa-adherent microbiota in CRC patients and healthy individuals.

### Microbial Composition of Intestinal Lumen in CRC Patients and Healthy Individuals

The intestinal lumen microbiota of CRC patients could be differentiated from healthy individuals according to unweighted Unifrac PCA analysis (Figure 2D). The families Erysipelotrichaceae (6.09% vs. 2.42%, *P* = 0.035), Prevotellaceae (1.46% vs. 1.14%, *P* = 0.035), Coriobacteriaceae (1.19% vs. 0.74%, *P* = 0.035), and Peptostreptococcaceae (0.89% vs. 0.45%, *P* = 0.035) were significantly enriched in CRC patients. Peptostreptococcaceae was also enriched in swab samples of CRC patients, whereas the relative abundance was higher compared to cancerous tissue. Genera *Peptostreptococcus* (Peptostreptococcaceae), *Porphyromonas*, *Mogibacterium*, *Anaerococcus*, *Slackia*, *Anaerotruncus*, *Collinsella* (Coriobacteriaceae), *Desulfovibrio*, *Eubacterium* and *Paraprevotella* were also more prevalent in patients compared to controls (Figure 4B).

Erysipelotrichaceae, Prevotellaceae, Coriobacteriaceae (*Collinsella*), *Peptostreptococcus*, and *Anaerotruncus* (Clostridiales), which were enriched in patients were classified as the key phylotypes that contribute to the separation of intestinal lumen microbiota structure in CRC patients and healthy individuals.

### Identification of Key OTUs Responsible for Structural Segregation of the Mucosa-associated Microbiota of Cancer and Control Samples

Sears and Pardoll proposed an Alpha-Bug hypothesis in a recent report–certain microbiome members that possessing unique virulence traits, such as enterotoxigenic *Bacteroides fragilis*, are not only directly pro-oncogenic but are capable of remodeling the microbiome as a whole, thus promoting mucosal immune responses and colonic epithelial cell changes and resulting in colon cancer [28]. We hypothesized that this Alpha-Bug potentially belongs to the mucosa-associated bacteria community. Firstly, LEfSe, a strict tool, was utilized to identify dominant OTUs. We found six dominant OTUs, which were all reduced in cancerous tissue and these key contributors belong to *Faecalibacterium*, *Dorea*, *uncultured Ruminococcus* sp., *Ruminococcus gnavus*, Lachnospiracea, and Peptostreptococcaceae. We generated a PLS-DA model was generated to find more OTUs that potentially contribute to the separation. OTUs that were differentially distributed were selected according to their variable importance in projection (VIP). A total of 27 OTUs with VIP>2 were identified as being relatively important contributors (four of these were enriched in cat; the others were reduced). These were members of Lachnospiracea (14), Bacteroidaceae (6), Ruminococcaceae (6), and Peptostreptococcaceae (1); all exhibited significant differences between cat and pa10t (*P*<0.05, Mann-Whitney test). Two additional OTUs closely related to *Ruminococcus gnavus* and 4 OTUs belonging to genus *Faecalibacterium* were also found to be reduced in cancerous tissue.

In addition, this analysis was performed using mucosa-adherent bacterial samples. Two dominant OTUs for each of the *Peptostreptococcus* sp. and *Parvimonas* sp. were enriched over 100 fold in CRC patients. One OTU related to *Bacteroides caccae* and one related to *Clostridium* sp. were also enriched. Two OTUs belonging to *Faecalibacterium* and *Blautia* were significantly reduced in patients. We selected 69 OTUs with VIP>2 that were important contributors according to PLS-DA, and 64 of them were significantly different between CRC patients and controls. Among them, six OTUs belonging to genus *Faecalibacterium* and six OTUs belonging to genus *Blautia* were reduced in patients with CRC. Additionally, two OTUs related to *Fusobacterium varium*, one OTU related to *Bacteroides xylanisolvens*, and one OTU related to *Dialister pneumosintes* were highly enriched in patients with CRC. Two additional OTUs related to *Peptostreptococcus sp.* and *Parvimonas sp.* were enriched in patients with CRC.

## Discussion

We speculated that the mucosa-associated microbiota primarily acts through direct interaction with the host and that intestinal lumen microbiota primarily acts through cometabolism or metabolic exchange with the host. We utilized barcoded multiplexed-454 pyrosequencing to compare the bacterial composition of cancerous tissue and intestinal lumen of patients with CRC to those of healthy controls. We also investigated the mucosa-adherent microbial composition by using rectal swab samples because the bacterial community is potentially altered by following bowel cleansing. We found that the structure of microbiota in cancerous tissue differs significantly differs from that of the intestinal lumen. The relative abundance of dominant phyla Firmicutes, Bacteroidetes, and Proteobacteria and dominant genera *Bacteroides*, *Streptococcus*, and *Pseudobutyrivibrio* were all different. Firmicutes, which has been demonstrated to enhance energy harvest from diet, was highly enriched in intestinal lumen [29], [30], [31]. Moreover, the predominant genus *Pseudobutyrivibrio* exhibited butyrate as a principal metabolite, as well as lactic acid and formic acid [32]. In contrast, Bacteroidetes, which is highly enriched in mucosa, may be primarily involved in interactions with the intestine [33]. The highly enriched major Gram-negative bacteria Proteobacteria in mucosa, with an outer membrane composed of lipopolysaccharides, potentially exhibits direct interaction with intestinal cells through bacterial secretion systems such as T2SS or T3SS [34], [35]. Additionally, enriched Fusobacteria and Synergistetes in mucosa also infects intestinal tissue [36], [37], [38]. As expected, our findings indicated that the structure of mucosa-adherent microbiota was more similar to tissue microbiota. Mucosa-adherent microbial structures also exhibited similarity with lumen microbiota; this is partially due to the unavoidable feces crossover on the swabs. We postulated that the swab microflora represents a combination of fecal microflora and a mucosa population less closely attached, whereas the tissue microbiota represents closely colonized bacteria. Unweighted Unifrac PCA analysis confirmed this result. Overall microbial structures were similar between cancerous tissue and noncancerous tissue. The intestinal lumen microbiota and mucosa-adherent microbiota were structurally separated in CRC patients compared to matched microbiota in healthy individuals.

Sears and Pardoll have proposed an Alpha-Bug hypothesis–certain microbiomes members not only are directly pro-oncogenic but are capable of remodeling the microbiome as a whole to promote cancer progression. We hypothesized that there are also certain microbiomes that can protect against pathogens and prevent the progression of cancer; for example, the segmented filamentous bacteria found in mouse intestine induce inflammation and protects against pathogens [39], [40]. Pyrosequencing data indicate *Faecalibacterium* is significantly less abundant in cancerous tissue compared to normal tissue. This finding was confirmed in mucosa-adherent microbiota of CRC patients compared to healthy controls. Additionally, four OTUs that were identified as key contributors to differentiate the microbial structures of tumor and normal tissue were significantly reduced. Moreover, six OTUs identified as key contributors to differentiate mucosa-adherent microbial structure of CRC patients and healthy individuals were significantly reduced. These results demonstrated that *Faecalibacterium* are negatively correlated to CRC. Sokol *et al*. reported that *F. prausnitzii*, the main species of *Faecalibacterium*, exhibits an anti-inflammatory effects on colitis by blocking NF-κB expression and IL-8 secretion [41]. Furthermore, *F. prausnitzii* induces colonization resistance against pathogens [42]. We hypothesized that *Faecalibacterium* plays a probiotic role in CRC. Interestingly, we found that three OTUs closely related to *Ruminococcus gnavus* are significantly reduced in cancerous tissue. *R. gnavus* produces an antibacterial peptide that protects hosts against pathogens [43]. Moreover, the amount of the probiotic *Bifidobacterium*, which counteracts pathogen colonization by competing for adhesion sites and secreting antibacterial peptides [44], was significantly reduced in CRC patients. In addition, *Fusobacterium* is a key phylotype that is significantly enriched in swab samples of CRC patients and is positively associated with CRC. Two of the enriched OTUs identified as key contributors are closely related to *Fusobacterium varium*, which can induce ulcerative colitis [36], [45]. *Fusobacterium* was also enriched in tumor tissue, although this finding was not statistically significant (data not shown). Taken together, our findings indicate that *Fusobacterium* was closely associated with CRC. Two recently published reports confirmed thses results [46], [47]. *Porphyromonas*, which is affiliated with family Porphyromonadaceae, was also found in abundance in CRC patients. Although rarely reported in the intestine, *P. gingivalis*, a main species of *Porphyromonas*, penetrates periodontal tissue, disrupt the host cell activity, and alters the microbiota composition to induce periodontitis [48], [49], [50]. However, *Peptostreptococcus* is commensal bacteria that can infect multiple sites of the body including intestinal mucosa under immunosuppressed or traumatic conditions. These results suggest that it is possible that the microbiome in mucosa mainly plays its role by directly interacting with the host.

Metabolites and antigens produced by microflora of the intestine may play vital roles in influencing CRC risk by interacting with host metabolism and immunity [51], [52]. Regarding microbiota of the intestinal lumen, the predominant phylotypes in CRC patients*–*Erysipelotrichaceae, Prevotellaceae, and Coriobacteriaceae–are all associated with metabolic disorders or energy metabolism. Erysipelotrichaceae, Prevotellaceae, and Coriobacteriaceae are enriched in obese human and obese mouse, as well as in “Western diet” or high-fat diet associated mouse, and they are closely related to energy production or adiposity [53], [54], [55], [56], [57], [58]. Epidemiological studies have established a strong association between “Western diet” or obesity (and its related metabolic diseases) and colorectal cancer. It seems that the enrichment of certain members of lumen microbiota is the basis for the association between “Western diet” or obesity and colorectal cancer. Furthermore, the enriched bacteria *Desulfovibrio* reduces sulfate in order to produce hydrogen sulfide, which has been reported as a possible contributing risk factor of CRC [59], [60]. In addition, Wang *et al*. found that butyrate-producing bacteria in the feces of CRC patients were reduced [7], and that butyrate plays an important role in cancer prevention [61]. These discoveries suggest that intestinal lumen microflora potentially exert an important influence on CRC risk through cometabolism or metabolic exchange with the host.

In conclusion, our results suggest that intestinal microbiota are associated with CRC risk, and that intestinal lumen microflora potentially influence CRC risk via cometabolism or metabolic exchange with the host. It is possible that mucosa-associated microbiota affect CRC risk largely through direct interaction with the host. The Alpha-Bug hypothesis may be suggested as follows: certain microbiome members of mucosa-associated microbiota, are not only directly pro-oncogenic but are capable of remodeling the intestinal lumen microbiota as a whole to promote progression of colon cancer. Our results represent a comprehensive picture of the microbial structure of CRC patients and help to further elucidate CRC etiology. However, more detailed information concerning mucosa-associated microbiota and lumen microbiota is essential. Moreover, the exact mechanisms contributing to the underlying changes remain obscure. Thus future studies are warranted to explore CRC microbiota and the different roles of such microbiota in CRC progression.

## Supporting Information

Figure S1 **Rarefaction curves.** Rarefaction curves were calculated at 3% distance with pyrosequencing data in microbiota from groups of cat, pa2t, pa10t, stc, stp, swc and swp.
(PDF)

Figure S2 **Heatmap analyses of 200 most abundant genera in groups of cat, pa2t, pa10t, stc, stp, swc and swp.** The y axis is a neighbor-joining phylogenetic tree, each row is a different phylotype. The abundance plot shows the proportion of 16S rRNA gene pyrosequences in each group.
(PDF)

Table S1 **Pyrosequencing data and estimator index of each sample.**
(DOC)

Table S2 **Phylotypes significantly different between microbiota of the intestinal lumen and cancerous tissue in CRC patients.**
(DOC)
