# Supplementary figures and images for: Human Intestinal Lumen and Mucosa-Associated Microbiota in Patients with Colorectal Cancer
Source: PLoS One. 2012 Jun 28;7(6):e39743. doi: 10.1371/journal.pone.0039743 (PMC3386193; doi:10.1371/journal.pone.0039743)

Number of OTUs Observed

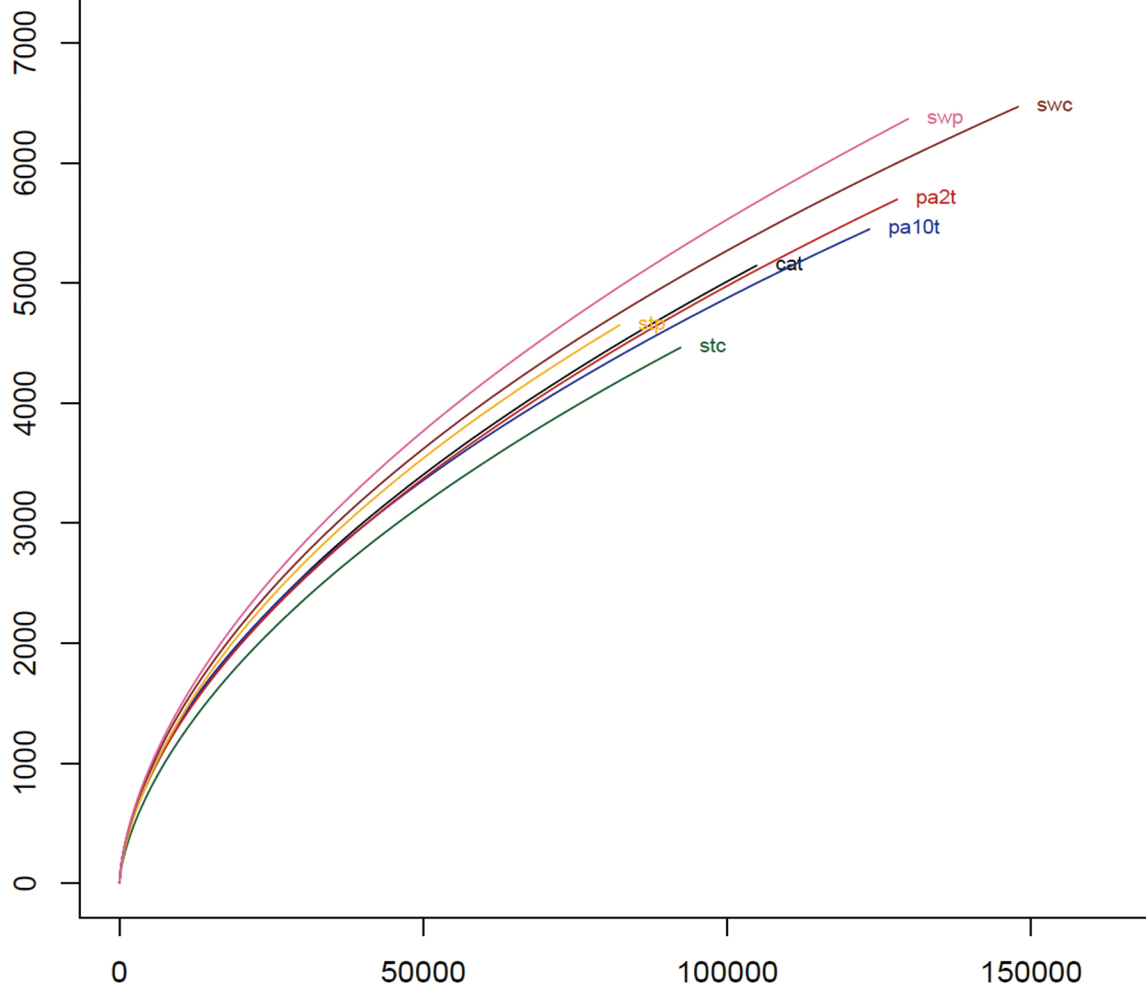

Number of Reads Sampled

Supplement: Figure S1 — Rarefaction curves. Rarefaction curves were calculated at 3% distance with pyrosequencing data in microbiota from groups of cat, pa2t, pa10t, stc, stp, swc and swp. (PDF) [file pone.0039743.s001.pdf]

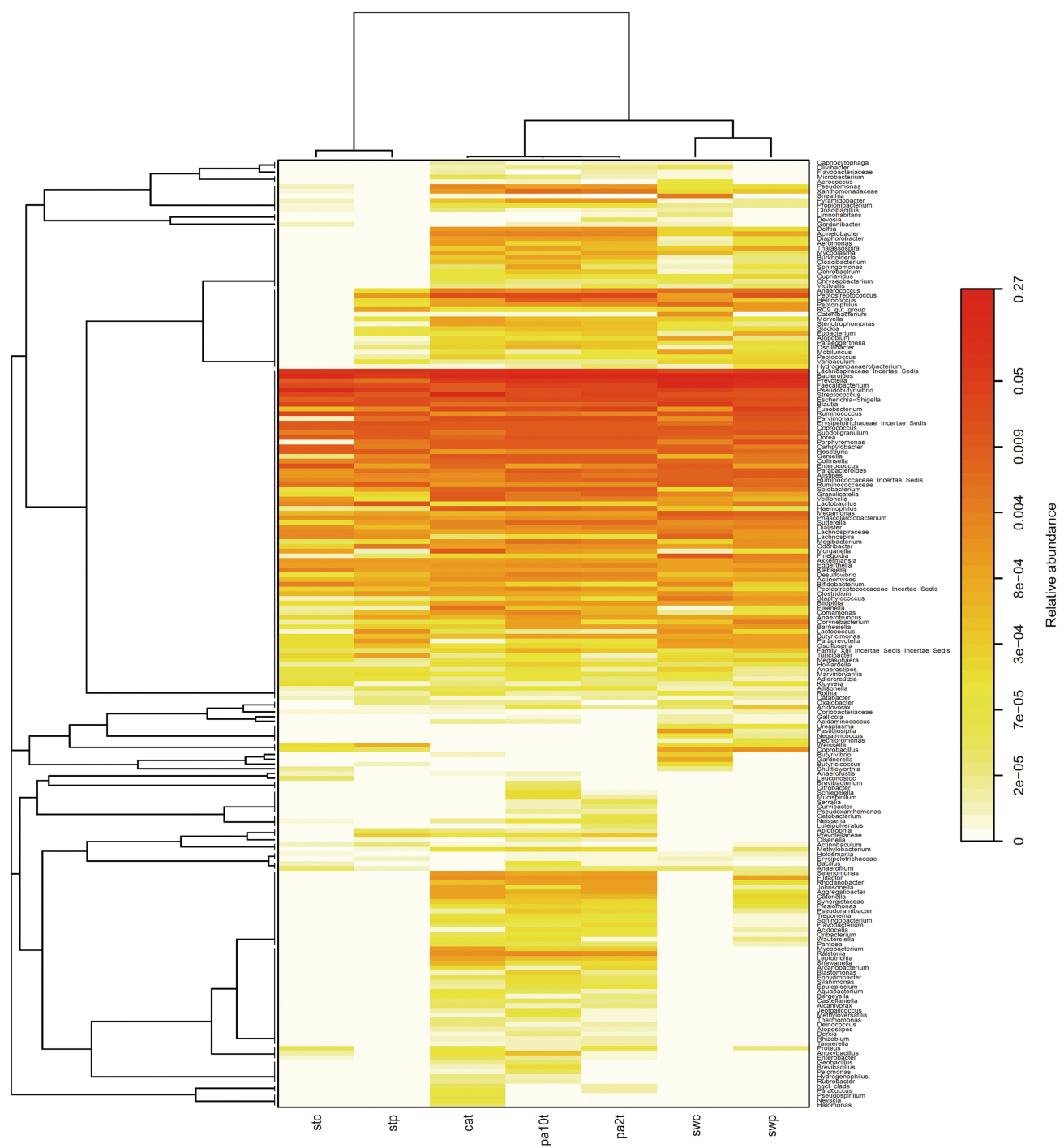

Supplement: Figure S2 — Heatmap analyses of 200 most abundant genera in groups of cat, pa2t, pa10t, stc, stp, swc and swp. The y axis is a neighbor-joining phylogenetic tree, each row is a different phylotype. The abundance plot shows the proportion of 16S rRNA gene pyrosequences in each group. (PDF) [file pone.0039743.s002.pdf]
