# Supplementary material for: Human Intestinal Lumen and Mucosa-Associated Microbiota in Patients with Colorectal Cancer
Source: PLoS One. 2012 Jun 28;7(6):e39743. doi: 10.1371/journal.pone.0039743 (PMC3386193; doi:10.1371/journal.pone.0039743)
Supplement: Table S1 — Pyrosequencing data and estimator index of each sample. (DOC) [file pone.0039743.s003.doc]

**Table S1.** Pyrosequencing data and estimator index of each sample

| Sample  ID | Total  sequences | OTUs | Chao | Shannon |  | Sample  ID | Total  sequences | OTUs | Chao | Shannon |
| --- | --- | --- | --- | --- | --- | --- | --- | --- | --- | --- |
| cat1 | 2988 | 380 | 899 | 4.19 |  | stc15 | 4487 | 422 | 1041 | 3.83 |
| cat2 | 4003 | 341 | 856 | 3.95 |  | stc16 | 4189 | 366 | 862 | 3.4 |
| cat3 | 3870 | 221 | 417 | 3.08 |  | stc17 | 5229 | 235 | 747 | 2.19 |
| cat4 | 4197 | 462 | 1199 | 4.35 |  | stc18 | 4020 | 325 | 802 | 3.18 |
| cat5 | 4372 | 581 | 1483 | 4.88 |  | stc19 | 4588 | 393 | 939 | 3.75 |
| cat6 | 4829 | 341 | 704 | 3.65 |  | stc20 | 4812 | 402 | 925 | 3.37 |
| cat7 | 3775 | 216 | 440 | 2.08 |  | stc21 | 4179 | 405 | 1065 | 3.85 |
| cat8 | 4274 | 348 | 804 | 3.94 |  | stc22 | 3861 | 428 | 948 | 4.14 |
| cat9 | 3456 | 386 | 890 | 3.72 |  | stp1 | 3871 | 541 | 1164 | 4.84 |
| cat10 | 3008 | 476 | 1058 | 4.66 |  | stp2 | 3967 | 430 | 990 | 4.06 |
| cat11 | 2331 | 292 | 664 | 4.13 |  | stp3 | 3667 | 329 | 999 | 3.54 |
| cat12 | 3806 | 301 | 596 | 3.48 |  | stp4 | 4296 | 455 | 934 | 3.87 |
| cat18 | 2272 | 418 | 984 | 4.95 |  | stp5 | 3562 | 410 | 969 | 4.05 |
| cat19 | 4832 | 454 | 946 | 3.76 |  | stp6 | 3672 | 355 | 776 | 4.1 |
| cat20 | 4911 | 298 | 587 | 3.16 |  | stp7 | 4131 | 380 | 986 | 3.64 |
| cat34 | 4284 | 283 | 678 | 3.24 |  | stp8 | 4363 | 373 | 785 | 3.48 |
| cat37 | 5019 | 363 | 1499 | 3.37 |  | stp9 | 4004 | 263 | 607 | 2.41 |
| cat38 | 4895 | 402 | 982 | 3.81 |  | stp10 | 3390 | 481 | 964 | 4.49 |
| cat39 | 3102 | 195 | 413 | 2.78 |  | stp11 | 4269 | 422 | 1036 | 3.96 |
| cat40 | 3830 | 350 | 813 | 3.57 |  | stp12 | 3859 | 465 | 1010 | 4.24 |
| cat41 | 5461 | 703 | 1410 | 4.88 |  | stp13 | 2823 | 384 | 918 | 4.2 |
| cat42 | 4881 | 388 | 1021 | 3.94 |  | stp14 | 3088 | 377 | 796 | 3.71 |
| cat43 | 4579 | 386 | 796 | 3.53 |  | stp15 | 3662 | 466 | 1150 | 4.37 |
| cat44 | 2340 | 270 | 605 | 3.8 |  | stp16 | 3728 | 356 | 861 | 3.65 |
| cat45 | 4514 | 451 | 968 | 4.32 |  | stp17 | 4930 | 395 | 886 | 3.46 |
| cat46 | 2026 | 187 | 417 | 3.58 |  | stp20 | 4987 | 419 | 939 | 3.86 |
| cat47 | 2863 | 230 | 510 | 3 |  | stp34 | 3801 | 414 | 1107 | 3.88 |
| pa2t1 | 3303 | 431 | 711 | 3.52 |  | stp35 | 4011 | 385 | 969 | 3.68 |
| pa2t2 | 5296 | 446 | 1231 | 4.37 |  | stp36 | 4212 | 447 | 962 | 4.29 |
| pa2t3 | 5044 | 365 | 865 | 3.67 |  | swc1 | 5287 | 621 | 1299 | 4.4 |
| pa2t4 | 5023 | 472 | 1241 | 4.33 |  | swc2 | 4199 | 362 | 964 | 3.72 |
| pa2t5 | 5285 | 536 | 1243 | 4.54 |  | swc3 | 4883 | 447 | 1048 | 3.94 |
| pa2t6 | 5145 | 554 | 1015 | 4.46 |  | swc4 | 4321 | 446 | 860 | 4.49 |
| pa2t7 | 2833 | 345 | 753 | 3.49 |  | swc5 | 4015 | 341 | 711 | 3.57 |
| pa2t8 | 4819 | 405 | 945 | 4.16 |  | swc6 | 4128 | 335 | 660 | 3.07 |
| pa2t9 | 4638 | 452 | 1084 | 4.23 |  | swc7 | 4424 | 419 | 891 | 4.27 |
| pa2t10 | 4810 | 769 | 1019 | 4.64 |  | swc8 | 4920 | 397 | 845 | 4.12 |
| pa2t11 | 2402 | 118 | 746 | 4.15 |  | swc9 | 4425 | 347 | 804 | 3.66 |
| pa2t12 | 4852 | 334 | 866 | 3.75 |  | swc10 | 3794 | 500 | 1196 | 4.54 |
| pa2t18 | 3270 | 366 | 837 | 4.27 |  | swc11 | 4155 | 350 | 849 | 3.58 |
| pa2t19 | 4951 | 387 | 756 | 3.48 |  | swc12 | 3930 | 414 | 908 | 3.48 |
| pa2t20 | 4569 | 450 | 1034 | 3.84 |  | swc13 | 4337 | 378 | 801 | 3.3 |
| pa2t34 | 5118 | 497 | 1135 | 4.17 |  | swc14 | 4604 | 524 | 1210 | 4.44 |
| pa2t37 | 4862 | 413 | 1010 | 3.74 |  | swc15 | 4599 | 439 | 1206 | 4.09 |
| pa2t38 | 5256 | 480 | 1242 | 3.99 |  | swc16 | 3897 | 322 | 658 | 3.42 |
| pa2t39 | 5327 | 358 | 665 | 3 |  | swc17 | 4977 | 525 | 1020 | 4.45 |
| pa2t40 | 4229 | 364 | 829 | 3.89 |  | swc18 | 3463 | 459 | 1190 | 4.52 |
| pa2t41 | 4826 | 466 | 1366 | 4.53 |  | swc19 | 4083 | 491 | 1021 | 4.73 |
| pa2t42 | 6442 | 359 | 1095 | 3.89 |  | swc20 | 3892 | 432 | 1161 | 4.29 |
| pa2t43 | 3995 | 431 | 1371 | 5.09 |  | swc21 | 5088 | 386 | 891 | 3.94 |
| pa2t44 | 5613 | 393 | 951 | 3.5 |  | swc22 | 3883 | 449 | 989 | 4.01 |
| pa2t45 | 5727 | 528 | 1358 | 4.34 |  | swc23 | 4408 | 374 | 694 | 3.46 |
| pa2t46 | 5534 | 385 | 1256 | 4.22 |  | swc24 | 4033 | 412 | 1044 | 3.55 |
| pa2t47 | 4652 | 406 | 829 | 3.84 |  | swc25 | 3678 | 423 | 1162 | 4.05 |
| pa10t1 | 3486 | 302 | 788 | 4.26 |  | swc26 | 4109 | 483 | 1072 | 4.26 |
| pa10t2 | 4772 | 442 | 1100 | 4.34 |  | swc27 | 4356 | 399 | 938 | 3.49 |
| pa10t3 | 4721 | 366 | 918 | 3.91 |  | swc28 | 4796 | 425 | 839 | 4.32 |
| pa10t4 | 4641 | 496 | 1059 | 4.22 |  | swc29 | 4642 | 387 | 970 | 3.38 |
| pa10t5 | 5498 | 521 | 1306 | 4.46 |  | swc30 | 4700 | 334 | 697 | 4.09 |
| pa10t6 | 3902 | 460 | 1171 | 4.98 |  | swc31 | 5150 | 455 | 953 | 4 |
| pa10t7 | 2335 | 316 | 653 | 4.6 |  | swc32 | 4405 | 488 | 1267 | 4.41 |
| pa10t8 | 4336 | 397 | 872 | 4.42 |  | swc33 | 3825 | 365 | 789 | 4.04 |
| pa10t9 | 4691 | 478 | 895 | 4.05 |  | swc34 | 4462 | 405 | 1146 | 3.5 |
| pa10t10 | 7974 | 504 | 1824 | 4.8 |  | swp1 | 3261 | 482 | 1065 | 4.73 |
| pa10t11 | 613 | 353 | 326 | 3.78 |  | swp2 | 3568 | 333 | 708 | 3.82 |
| pa10t12 | 4684 | 392 | 565 | 3.8 |  | swp3 | 4927 | 325 | 617 | 2.8 |
| pa10t18 | 4324 | 405 | 760 | 3.7 |  | swp4 | 5433 | 521 | 1100 | 4.49 |
| pa10t19 | 5892 | 349 | 862 | 3.37 |  | swp5 | 3755 | 469 | 1029 | 4.51 |
| pa10t20 | 2978 | 391 | 1047 | 4.49 |  | swp6 | 4009 | 388 | 888 | 3.52 |
| pa10t34 | 5541 | 459 | 1025 | 4.29 |  | swp7 | 4163 | 483 | 1019 | 4.18 |
| pa10t37 | 4630 | 450 | 900 | 3.46 |  | swp8 | 4241 | 389 | 845 | 3.95 |
| pa10t38 | 5373 | 531 | 1134 | 3.96 |  | swp9 | 4517 | 272 | 616 | 2.6 |
| pa10t39 | 2447 | 309 | 665 | 4.64 |  | swp10 | 3353 | 403 | 828 | 4.39 |
| pa10t40 | 3971 | 429 | 893 | 4.01 |  | swp11 | 3968 | 458 | 1126 | 4.33 |
| pa10t41 | 4698 | 584 | 892 | 4.36 |  | swp12 | 3988 | 382 | 1034 | 3.85 |
| pa10t42 | 5121 | 467 | 883 | 3.8 |  | swp13 | 3691 | 396 | 994 | 4.04 |
| pa10t43 | 5060 | 636 | 933 | 3.88 |  | swp14 | 3854 | 344 | 1079 | 3.58 |
| pa10t44 | 5326 | 388 | 977 | 3.7 |  | swp15 | 4969 | 451 | 1065 | 4.18 |
| pa10t45 | 5613 | 525 | 1122 | 4.42 |  | swp16 | 3965 | 446 | 1095 | 4.1 |
| pa10t46 | 4477 | 506 | 930 | 4.01 |  | swp17 | 3593 | 361 | 807 | 3.83 |
| pa10t47 | 6116 | 372 | 992 | 3.77 |  | swp18 | 5008 | 362 | 611 | 4.04 |
| stc1 | 3851 | 219 | 446 | 3.01 |  | swp19 | 3553 | 347 | 734 | 3.7 |
| stc2 | 3813 | 435 | 966 | 4.15 |  | swp20 | 4390 | 284 | 561 | 2.37 |
| stc3 | 3364 | 467 | 1146 | 4.58 |  | swp21 | 4929 | 478 | 859 | 4.63 |
| stc4 | 3889 | 419 | 898 | 4.18 |  | swp22 | 4092 | 288 | 550 | 2.93 |
| stc5 | 4207 | 438 | 1090 | 3.89 |  | swp23 | 4369 | 439 | 882 | 4.33 |
| stc6 | 4722 | 369 | 783 | 3.26 |  | swp24 | 3829 | 438 | 966 | 4.3 |
| stc7 | 4605 | 402 | 1014 | 3.44 |  | swp25 | 3999 | 486 | 967 | 4.38 |
| stc8 | 4496 | 453 | 1093 | 3.91 |  | swp26 | 3495 | 439 | 1426 | 4.18 |
| stc9 | 3387 | 475 | 995 | 4.44 |  | swp27 | 3653 | 492 | 1147 | 4.5 |
| stc10 | 4341 | 492 | 1193 | 4.07 |  | swp29 | 3276 | 444 | 992 | 4.15 |
| stc11 | 3720 | 346 | 1057 | 3.49 |  | swp30 | 3470 | 503 | 1126 | 4.7 |
| stc12 | 3838 | 391 | 1016 | 3.83 |  | swp31 | 3967 | 400 | 975 | 3.87 |
| stc13 | 4587 | 401 | 1184 | 3.22 |  | swp32 | 4795 | 470 | 981 | 4.39 |
| stc14 | 4148 | 440 | 951 | 4.16 |  | swp33 | 3675 | 370 | 1093 | 3.9 |

The number of OTUs, richness estimator Chao, and diversity estimator Shannon were calculated at 3% distance.

Samples cat1, pa2t1, pa10t1, stp1 and swp1 are from the same patient. The rest sample identification number for CRC patient may be deduced by analogy.
