# Supplementary material for: Human Intestinal Lumen and Mucosa-Associated Microbiota in Patients with Colorectal Cancer
Source: PLoS One. 2012 Jun 28;7(6):e39743. doi: 10.1371/journal.pone.0039743 (PMC3386193; doi:10.1371/journal.pone.0039743)
Supplement: Table S2 — Phylotypes significantly different between microbiota of the intestinal lumen and cancerous tissue in CRC patients. (DOC) [file pone.0039743.s004.doc]

**Table S2.** Phylotypes significantly different between microbiota of the intestinal lumen and cancerous tissue in CRC patients.

| Taxonomic Rank | | Relative abundance (%)# | | p-value |
| --- | --- | --- | --- | --- |
|  |  | cat | stp |  |
| phylum | Actinobacteria |  |  |  |
| class | Actinobacteridae(subclass) |  |  |  |
| order | Actinomycetales | 0.94 | 0.14 | 0.033 |
| family | Mycobacteriaceae | 0.43 | 0 | 0.004 |
| genus | *Mycobacterium* | 0.43 | 0 | 0.004 |
| phylum | Bacteroidetes | 26.37 | 13.68 | 0.002 |
| class | Bacteroidia | 26.10 | 13.66 | 0.002 |
| order | Bacteroidales | 26.13 | 13.65 | 0.002 |
| family | Bacteroidaceae | 16.95 | 8.32 | <0.001 |
| genus | *Bacteroides* | 16.95 | 8.32 | <0.001 |
| family | Prevotellaceae |  |  |  |
| genus | *Paraprevotella* | 0.002 | 0.247 | 0.006 |
| genus | *Prevotella* | 6.11 | 0.88 | 0.011 |
| family | Porphyromonadaceae |  |  |  |
| genus | *Porphyromonas* | 1.16 | 0.77 | 0.024 |
| class | Flavobacteria | 0.196 | 0 | <0.001 |
| order | Flavobacteriales | 0.196 | 0 | <0.001 |
| family | Flavobacteriaceae | 0.196 | 0 | <0.001 |
| class | Sphingobacteria | 0.036 | 0 | 0.013 |
| order | Sphingobacteriales | 0.036 | 0 | 0.013 |
| family | Sphingobacteriaceae | 0.036 | 0 | 0.013 |
| phylum | Firmicutes | 50.82 | 77.59 | <0.001 |
| class | Bacilli | 14.56 | 6.69 | 0.008 |
| order | Bacillales | 2.02 | 0.65 | <0.001 |
| genus | *Gemella* | 1.97 | 0.65 | <0.001 |
| order | Lactobacillales | 12.53 | 6.03 | 0.012 |
| family | Carnobacteriaceae | 1.64 | 0.045 | <0.001 |
| genus | *Granulicatella* | 1.63 | 0.045 | <0.001 |
| family | Leuconostocaceae | 0 | 0.123 | 0.001 |
| genus | *Weissella* | 0 | 0.123 | 0.001 |
| family | Streptococcaceae | 10.20 | 2.80 | 0.003 |
| genus | *Streptococcus* | 10.19 | 2.45 | 0.001 |
| family | Lactobacillaceae | 0.022 | 2.88 | 0.009 |
| genus | *Lactobacillus* | 0.022 | 2.88 | 0.009 |
| class | Clostridia | 33.74 | 64.05 | <0.001 |
| order | Clostridiales | 33.74 | 64.05 | <0.001 |
| family | Peptostreptococcaceae | 4.07 | 0.89 | <0.001 |
| genus | *Peptostreptococcus* | 3.39 | 0.29 | <0.001 |
| genus | *Filifactor* | 0.35 | 0 | 0.007 |
| family | Lachnospiraceae | 17.11 | 46.66 | <0.001 |
| genus | *Roseburia* | 0.23 | 2.08 | <0.001 |
| genus | *Blautia* | 0.61 | 3.70 | <0.001 |
| genus | *Lachnospira* | 0.005 | 0.42 | 0.002 |
| genus | *Pseudobutyrivibrio* | 1.77 | 7.74 | 0.004 |
| genus | *Johnsonella* | 0.15 | 0 | 0.007 |
| genus | *Dorea* | 0.73 | 1.68 | 0.014 |
| genus | *Coprococcus* | 0.90 | 1.50 | 0.025 |
| family | Veillonellaceae | 2.87 | 0.68 | 0.041 |
| genus | *Selenomonas* | 0.24 | 0 | 0.002 |
| genus | *Veillonella* | 1.85 | 0.022 | 0.025 |
| genus | *Dialister* | 0.49 | 0.086 | 0.014 |
| family | Clostridiaceae | 0.128 | 0.300 | 0.033 |
| genus | *Clostridium* | 0.128 | 0.300 | 0.033 |
| family | Ruminococcaceae | 4.24 | 13.34 | <0.001 |
| class | Erysipelotrichi |  |  |  |
| order | Erysipelotrichales |  |  |  |
| family | *Erysipelotrichaceae* |  |  |  |
| genus | *Turicibacter* | 0.005 | 0.145 | 0.008 |
| genus | *Solobacterium* | 0.852 | 0.214 | 0.028 |
| phylum | Fusobacteria | 4.97 | 0.47 | <0.001 |
| order | Fusobacteriales | 4.97 | 0.47 | <0.001 |
| genus | *Leptotrichia* | 0.154 | 0 | 0.04 |
| family | Fusobacteriaceae | 4.568 | 0.471 | <0.001 |
| genus | *Fusobacterium* | 4.567 | 0.471 | <0.001 |
| phylum | Proteobacteria | 14.51 | 5.57 | 0.004 |
| class | Alphaproteobacteria | 0.136 | 0.001 | 0.013 |
| order | Rhodospirillales | 0.034 | 0 | 0.04 |
| family | Rhodospirillaceae | 0.033 | 0 | 0.04 |
| genus | *Thalassospira* | 0.032 | 0 | 0.04 |
| class | Betaproteobacteria | 2.95 | 0.32 | 0.001 |
| order | Burkholderiales | 2.11 | 0.32 | 0.002 |
| family | Comamonadaceae | 1.01 | 0.10 | <0.001 |
| genus | *Delftia* | 0.34 | 0 | <0.001 |
| family | Alcaligenaceae |  |  |  |
| genus | *Sutterella* | 0.42 | 0.17 | 0.034 |
| family | Burkholderiaceae | 0.64 | 0 | 0.001 |
| genus | *Burkholderia* | 0.067 | 0 | 0.007 |
| genus | *Ralstonia* | 0.55 | 0 | 0.023 |
| genus | *Cupriavidus* | 0.016 | 0 | 0.04 |
| order | Neisseriales | 0.83 | 0.002 | 0.005 |
| family | Neisseriaceae | 0.83 | 0.002 | 0.005 |
| class | Deltaproteobacteria | 0.37 | 0.13 | 0.006 |
| order | Desulfovibrionales | 0.37 | 0.13 | 0.006 |
| family | Desulfovibrionaceae | 0.37 | 0.13 | 0.006 |
| genus | *Bilophila* | 0.14 | 0.04 | 0.001 |
| class | Gammaproteobacteria | 9.51 | 4.48 | 0.009 |
| order | Aeromonadales | 0.20 | 0 | 0.04 |
| family | Aeromonadaceae | 0.20 | 0 | 0.04 |
| genus | *Aeromonas* | 0.20 | 0 | 0.04 |
| order | Alteromonadales | 0.056 | 0 | 0.04 |
| family | Shewanellaceae | 0.056 | 0 | 0.04 |
| genus | *Shewanella* | 0.056 | 0 | 0.04 |
| order | Enterobacteriales |  |  |  |
| family | Enterobacteriaceae |  |  |  |
| genus | *Morganella* | 1.58 | 0.003 | 0.045 |
| order | Pasteurellales | 2.25 | 0.007 | <0.001 |
| family | Pasteurellaceae | 2.25 | 0.007 | <0.001 |
| genus | *Haemophilus* | 2.12 | 0.007 | <0.001 |
| order | Pseudomonadales | 1.23 | 0 | <0.001 |
| family | Moraxellaceae | 0.68 | 0 | <0.001 |
| genus | *Acinetobacter* | 0.66 | 0 | <0.001 |
| family | Pseudomonadaceae | 0.53 | 0 | 0.001 |
| genus | *Pseudomonas* | 0.53 | 0 | 0.001 |
| order | Xanthomonadales | 0.746 | 0.003 | <0.001 |
| family | Xanthomonadaceae | 0.72 | 0.003 | <0.001 |
| genus | *Rhodanobacter* | 0.095 | 0 | 0.004 |
| genus | *Stenotrophomonas* | 0.188 | 0.001 | 0.023 |
| phylum | Synergistetes | 0.142 | 0 | 0.002 |
| class | Synergistia | 0.142 | 0 | 0.002 |
| order | Synergistales | 0.142 | 0 | 0.002 |
| family | Synergistaceae | 0.142 | 0 | 0.002 |

Statistical analysis was performed by Mann-Whitney test.

#Relative abundance were shown as mean. Data had no statistically significant difference were not shown.
